# Are New β-Lactam/β-Lactamase Inhibitor Combinations Promising Against Carbapenem-Resistant *K. pneumoniae* Isolates?

**DOI:** 10.3390/pathogens14030220

**Published:** 2025-02-24

**Authors:** Ayşe Nur Ceylan, Selda Kömeç, Kamuran Şanlı, Beyza Öncel, Mehmet Akif Durmuş, Abdurrahman Gülmez

**Affiliations:** 1 Department of Medical Microbiology, Basaksehir Çam and Sakura City Hospital, University of Health Science, Istanbul 34480, Türkiye; 2 Medical Microbiology Laboratory, Aydın Ataturk State Hospital, Aydın 09020, Türkiye

**Keywords:** carbapenem-resistant *Klebsiella pneumoniae*, ceftazidime/avibactam, meropenem/vaborbactam, OXA-48, NDM, KPC

## Abstract

Background/Objectives: Carbapenem-resistant *Klebsiella pneumoniae* (CRKP) infections present a significant clinical challenge due to limited therapeutic options and high transmission potential. This study aimed to identify the resistance genes associated with carbapenemase production in CRKP isolates and evaluate the in vitro activity of ceftazidime/avibactam (CZA) and meropenem/vaborbactam (MEV), among other β-lactam/β-lactamase inhibitor combinations. Methods: Between October 2021 and June 2022, a total of 504 CRKP isolates were grown from patient samples in intensive care units. When duplicate patient samples were removed, the remaining 89 isolates were included in the study. Bacterial identification and antimicrobial susceptibility testing were per-formed using MALDI-TOF, Phoenix M50, and disk diffusion methods, following EUCAST guidelines. PCR analyses identified carbapenemase genes such as OXA-48, NDM, and KPC. Results: The most prevalent carbapenemase gene was OXA-48 (79.8%), followed by NDM (21.4%) and KPC (17.9%). The susceptibility rate to CZA was 82.0%, significantly higher than MEV (10.1%). All isolates were resistant to piperacillin/tazobactam and ceftolozane/tazobactam. Among MEV-resistant isolates, most carried the OXA-48 gene, while NDM was common in CZA-resistant isolates. Conclusions: CZA demonstrates high efficacy against OXA-48-producing CRKP, making it a viable treatment option in settings where OXA-48 predominates. The limited activity of MEV in this study underscores the need for molecular surveillance of resistance mechanisms to guide empirical therapy.

## 1. Introduction

Carbapenem-resistant *Enterobacterales* (CRE) present a significant risk of morbidity and mortality among hospitalized patients. *Klebsiella pneumoniae*, part of the *Enterobacterales* family, is particularly concerning because it can cause severe infections in immunocompromised individuals, those with underlying health conditions, and patients undergoing invasive procedures. This bacterium is a common pathogen in healthcare settings and is associated with various infections, including pneumonia, bloodstream infections, urinary tract infections (UTI), and surgical site infections [1,2].

According to the 2016 surveillance data from the National Antimicrobial Resistance Surveillance System (UAMDSS), the resistance rates for invasive *K. pneumoniae* isolates were as follows: 40.1% for imipenem and meropenem from the carbapenem group, and 48.9% for ertapenem [3]. Additionally, the 2021 data from the Central Asia and Eastern Europe Antimicrobial Resistance Surveillance (CAESAR) reported carbapenem resistance rates in our country to be over 25% [4].

Different mechanisms, such as porin loss and efflux pumps, may also play a role in the development of resistance to antibiotics in Gram-negative bacteria. However, the most important mechanism in the development of resistance is β-lactamase production. The Ambler system classifies β-lactamases into four main classes based on their molecular structure: Class A, B, C, and D. Classes A, C, and D contain serine in their active sites, while Class B requires zinc to hydrolyze β-lactams. Carbapenems are resistant to certain enzymes in Class A, such as TEM, SHV, and CTX-M, while KPC and GES enzymes can hydrolyze carbapenems. Among the various types of KPC identified in *Enterobacterales*, KPC-2 and KPC-3 are the most common. Class B enzymes include metallo-β-lactamases that are capable of hydrolyzing carbapenems, such as VIM, IMP, and NDM. The most significant enzyme in Class C is AmpC, which is found chromosomally in many Gram-negative bacteria but is generally not considered to exhibit carbapenemase activity. Class D consists of OXA-type carbapenemases, with enzymes in the OXA-48 group being particularly associated with carbapenem resistance in the *Enterobacterales* family [5,6].

Among the mechanisms underlying carbapenem resistance in *K. pneumoniae*, the most significant is the production of carbapenemases, including class A (mainly KPC), class B metallo-β-lactamases (notably NDM), and certain class D OXA-48-like enzymes. Occurrence rates of these carbapenemases vary by geographical region [7]. OXA-48, for example, was first detected in Turkiye in 2001 in a *K. pneumoniae* isolate, marking its initial global identification [8]. Since then, the prevalence of the OXA-48 gene has continued to rise, and studies indicate that it remains the most common carbapenemase in Turkiye. Other carbapenemase genes, such as NDM, VIM, and IMP, are also observed, following OXA-48 in prevalence [9,10]. Detecting the genes responsible for carbapenemase production in bacteria isolated from hospitals is crucial for epidemiological purposes, as it aids in tracking the spread of resistant strains and informing infection control measures.

The increasing prevalence of CRE infections, particularly those caused by *K. pneumoniae*, presents a significant challenge for healthcare facilities due to limited treatment options and a high potential for transmission between patients, especially in intensive care units. Although ceftolozane/tazobactam and piperacillin/tazobactam are effective against most ESBL-producing organisms, they are inactive against carbapenemases such as KPC, certain class D enzymes, and metallo-β-lactamases [11].

Recent therapeutic strategies have introduced novel combinations of β-lactam antibiotics and β-lactamase inhibitors, such as ceftazidime/avibactam (CZA) and meropenem/vaborbactam (MEV). These combinations have shown promising activity against certain carbapenemase-producing organisms. Ceftazidime/avibactam is a combination of ceftazidime, a third-generation cephalosporin, and avibactam, a novel non-β-lactam/β-lactamase inhibitor. Avibactam acts as a potent β-lactamase inhibitor, neutralizing class A carbapenemases (notably KPC-2), extended-spectrum β-lactamases, class C cephalosporinases, and some class D carbapenemases. However, it is ineffective against metallo-β-lactamase-producing strains, and CZA-resistant KPC variants have been observed in recent years [12,13]. MEV (Vabomere™) has received U.S. approval for the treatment of complicated UTI, including pyelonephritis. Vaborbactam targets class A serine carbapenemases and enhances the activity of meropenem against KPC-producing *Enterobacterales*. While it displays potent in vitro activity against these Gram-negative isolates, it is inactive against OXA-48 [14].

Başakşehir Çam and Sakura City Hospital is the largest hospital in Istanbul, featuring a capacity of 2682 beds and consisting of eight separate hospitals. It has a total intensive care capacity of 465 patients, which includes 288 beds in adult intensive care units and 177 beds in pediatric intensive care units. All samples collected from both inpatients and outpatients are analyzed in the central laboratory. In this study, we aimed to determine the resistance genes that cause carbapenemase production in carbapenem-resistant *K. pneumoniae* isolates obtained from patient samples in the intensive care units of our hospital. Carbapenem-resistant *K. pneumoniae* poses a significant challenge in clinical settings due to its high resistance to a large number of antibiotics, and this poses a major problem in terms of patient treatment and infection control. In our study, we focused on determining the genetic mechanisms contributing to resistance formation by investigating the presence of different carbapenemase-producing genes such as KPC, NDM, VIM, and OXA. In addition to molecular analyses, we evaluated the in vitro efficacy of various β-lactam/β-lactamase inhibitor combinations such as CZA, MEV, piperacillin/tazobactam, and ceftolozane/tazobactam on resistant isolates. Our aim was to determine the suitability of these antibiotics for clinical use in our hospital population.

## 2. Materials and Methods

### 2.1. Selection of Isolates

The study focused on *K. pneumoniae* isolates that were resistant to carbapenems (ertapenem, meropenem, and imipenem). The isolates were collected from blood and tracheal aspirate samples of patients admitted to the intensive care unit at Başakşehir Çam and Sakura City Hospital in Istanbul, Turkiye, between October 2021 and June 2022. No distinction was made between colonization and infection in these samples. The samples that arrived at the medical microbiology laboratory were cultured under appropriate conditions and examined according to national guidelines [15,16]. To ensure the integrity of the study, isolates from repeated cultures of the same patient were excluded, with only the initial sample growths being considered. In addition, isolates showing susceptible, standard dosing regimen or susceptible, increased exposure to any carbapenem through antibiotic susceptibility testing were not included in the study.

### 2.2. Bacterial Identification and Antibiotic Susceptibility Test

Isolates grown on solid media were identified with MALDI-TOF Microflex LT/SH Smart MS (Bruker Daltonics, Bremen, Germany). Antibiotic susceptibility testing was conducted with the Phoenix M50 (BD Diagnostics, Sparks, MD, USA) using the NMIC kit (BD Diagnostics, Sparks, MD, USA) for Gram-negative bacteria. Since the NMIC kit did not contain CZA and MEV antibiotics, the Kirby–Bauer disk diffusion method was applied. Bacterial solutions with a turbidity of 0.5 McFarland were prepared and streaked in Mueller–Hinton medium (Biocell, Istanbul, Turkiye), and CZA (10-4 µg) and MEV (20-10 µg) disks (Oxoid Limited, Hampshire, UK) were placed and incubated. Zone diameters were evaluated the next day. The results of the antibiotic susceptibility tests were assessed according to the EUCAST breakpoints table (v.14.0) [17]. No phenotypic method was used to detect carbapenemase production.

In the medical microbiology laboratory, internal quality control studies are carried out regularly for both the automated microdilution system and the Kirby–Bauer disk diffusion method for *Escherichia coli* ATCC 25922 and *Klebsiella pneumoniae* ATCC 700603. This study was also conducted during our research, and the results were within the expected range.

### 2.3. Nucleic Acid Extraction

Nucleic acid isolation was performed using colonies grown on solid media. The Bio-Speedy^®^ Rapid Nucleic Acid Extraction Kit (Bioeksen, Istanbul, Turkiye) and the Zybio EXM3000 Nucleic Acid Isolation System (Zybio, Chongqing, China) were utilized in this process. The protocol was optimized according to the manufacturer’s instructions. Colonies growing on solid media were collected using a sterile loop. One to three colonies were transferred to 2 mL centrifuge tubes for each sample. Subsequently, 200 µL of Bio-Speedy^®^ STL-B buffer was added, and the mixture was vortexed at maximum speed for 10 s to ensure homogenization. Then, 200 µL of the prepared sample was pipetted into the first well of the extraction cartridge, followed by the addition of 15 µL of Proteinase K. The robotic system completed the lysis, binding, washing, and elution steps automatically. Purified nucleic acids were collected from the 6th well of the cartridge, transferred to nuclease-free centrifuge tubes, and stored at −20 °C.

### 2.4. PCR Analysis

To detect carbapenem resistance genes, the Bio-Speedy^®^ Carbapenem Resistance qPCR Kit (Bioeksen, Istanbul, Turkiye) was employed. This kit is designed to rapidly and sensitively identify carbapenem resistance-associated genes, including KPC, NDM, VIM, IMP, OXA-23, OXA-48, OXA-51, and OXA-58. PCR analyses were conducted using the Bio-Rad CFX96 Touch™ Real-Time PCR System (Bio-Rad, Hercules, CA, USA), and the amplification results were analyzed with Sigmoida Analysis Software v 8.6 (Bioeksen, Istanbul, Turkiye). Each qPCR reaction was set up with a final volume of 10 µL, consisting of 5 µL of 2X qPCR Mix, 2.5 µL of CRE or OXA Oligo Mix, and 2.5 µL of the nucleic acid template. Positive controls (PC) and negative controls (NTC) were included in every run to ensure the accuracy of the assay. The PCR cycle program began with reverse transcription at 52 °C for 3 min, followed by initial denaturation at 95 °C for 10 s. Subsequently, a touchdown protocol was applied, with the annealing temperature reduced by 1 °C per cycle from 67 °C to 56 °C over 12 cycles. This was followed by 35 cycles of fixed annealing/extension at 55 °C for 10 s.

Tests were repeated and confirmed for isolates with results that appeared inconsistent following antibiotic susceptibility testing and PCR studies.

## 3. Results

Between October 2021 and June 2022, our laboratory received a total of 58,796 samples from 8432 patients hospitalized in the intensive care units of our hospital. This included 53,734 blood culture samples and 5062 tracheal aspirate culture samples. We observed the growth of Gram-negative bacteria in 2979 blood cultures and 2320 tracheal aspirate cultures. Among these, *K. pneumoniae* accounted for 839 (28.2%) of the Gram-negative bacteria in blood cultures and 411 (17.7%) in tracheal aspirate cultures. Of the *K. pneumoniae* isolates, 504 were resistant to all carbapenems, including ertapenem, meropenem, and imipenem. We removed repeated samples from our analysis and only included the initial samples from each patient. Ultimately, 89 isolates were selected for the PCR study.

The study involved 89 *K. pneumoniae* isolates that were cultured from various clinical samples of patients hospitalized in intensive care units. All isolates exhibited resistance to carbapenems (ertapenem, meropenem, and imipenem) studied with the Phoenix NMIC kit. Among the *K. pneumoniae* isolates, 97.8% (87 isolates) were resistant to ceftolozane/tazobactam, while 100% (89 isolates) were resistant to piperacillin/tazobactam. The disc diffusion results for CZA and MEV revealed resistance rates of 16.9% (15 isolates) and 91.0% (81 isolates), respectively. The carbapenemase resistance genes detected in the isolates through PCR are presented in Table 1. Upon examining the frequency of these resistance genes, the most prevalent gene identified was OXA-48, found in a total of 73 isolates, either alone or in combination with other genes. This was followed by the NDM gene in 24 isolates and the KPC gene in 16 isolates. A total of 89 *K. pneumoniae* isolates were included in the study. Among these isolates, resistance genes showed varying distribution patterns. The OXA-48 gene was the most frequently detected, found in 56 isolates (62.9%). A total of 9 isolates (10.1%) co-harbored the OXA-48 and NDM genes, while the KPC gene was identified in another 9 isolates (10.1%). The NDM gene alone was detected in 8 isolates (9.0%). Additionally, co-occurrence of OXA-48 and KPC genes was observed in 5 isolates (5.6%), and a single isolate (1.1%) carried all three resistance genes: OXA-48, KPC, and NDM. Similarly, 1 isolate (1.1%) was found to harbor both KPC and NDM genes. OXA-23, OXA-51, and VIM genes were not detected. These results underscore the dominance of OXA-48 and the presence of various resistance gene combinations among the isolates (Table 1 and Table S1).

In our analysis of 80 isolates that are resistant to MEV, we found that 70 of them carried the OXA-48 gene. Among the 10 isolates without the OXA-48 gene, 9 had the NDM gene. The last isolate that was resistant to MEV only showed the presence of the KPC gene. In contrast, 8 of the 9 isolates that were susceptible to MEV contained only the KPC gene, while the remaining isolate carried only the OXA-48 gene. Looking at the 15 isolates resistant to CZA, we detected the NDM gene in all of them. Of the 73 isolates that were susceptible to CZA, we identified the following carbapenemase genes: OXA-48 in 56 isolates, KPC in 9 isolates, OXA-48 + KPC in 5, OXA-48 + NDM in 2, and NDM in 1 isolate (Table 2).

## 4. Discussion

Carbapenem-resistant *K. pneumoniae* strains pose a significant clinical challenge due to the limited treatment options available and their potential for rapid spread within healthcare settings. In this context, β-lactam/β-lactamase inhibitor combinations, such as CZA and MEV, have emerged as promising therapeutic options, offering new hope for treating these carbapenem-resistant isolates. The effectiveness of CZA and MEV in treating these infections varies significantly based on the underlying resistance genes present. An analysis of the resistance genes in the isolates revealed that OXA-48 was the most prevalent, found in 71 isolates (79.8%). This gene was present either alone or in combination with others. The next most common genes were NDM, identified in 19 isolates (21.4%), and KPC, detected in 16 isolates (17.9%) (see Table 1).

In a surveillance study conducted in Spain, the antibiotic susceptibility of *K. pneumoniae*—responsible for both intra-abdominal (n:165) and UTI (n:205)—was examined. The susceptibility of *K. pneumoniae* isolates to piperacillin/tazobactam was recorded as 66.6% for intra-abdominal infections and 69.7% UTI. The rates of extended-spectrum beta-lactamase (ESBL) production in these isolates were found to be 24.4% and 32.6%, respectively. Among the ESBL-positive isolates, piperacillin/tazobactam susceptibility was notably lower, with rates of less than 15% in intra-abdominal infections and less than 30% in UTI [18]. In a separate study conducted in Egypt in 2022, 180 *K. pneumoniae* isolates were analyzed, revealing that 119 of them were resistant to carbapenems. All carbapenem-resistant isolates were also found to be resistant to piperacillin/tazobactam [19]. Similarly, in our study, all tested isolates were resistant to piperacillin/tazobactam.

Ceftolozane/tazobactam susceptibility rates are typically reported among ESBL-producing *K. pneumoniae* isolates, while susceptibility data for carbapenem-resistant isolates are limited [20]. For instance, in a study conducted in Abu Dhabi, only 10% of the 60 CRE isolates were susceptible to ceftolozane/tazobactam [21]. Similarly, a study in Turkiye found that just 5% of carbapenem-resistant *K. pneumoniae* isolates demonstrated susceptibility to ceftolozane/tazobactam [22]. In our study, only 2 out of 89 carbapenem-resistant isolates (2.2%) were susceptible to the drug. When we look at the 2 isolates that were found to be susceptible to ceftolozane/tazobactam despite having carbapenemase production, we see that both have MIC values of 2 mg/L. There are only three dilutions for this antibiotic in the NMIC kit used (1, 2, and 4 mg/L), and three dilution wells may be insufficient. As stated in the “technical uncertainty” section of the EUCAST breakpoint table, there may be a dilution error in microdilution tests. In these isolates, when the MIC value is at the next dilution, the result changes categorically and becomes “resistant” [17]. Upon analyzing the two ceftolozane/tazobactam susceptible strains, the presence of OXA-48 was detected. It was found that CZA was susceptible while MEV was resistant. Given this information, the MIC result for ceftolozane/tazobactam may be erroneous.

In King et al.’s 2017 study, the effectiveness of CZA on CRE isolates was examined. The study included 60 patients, most of whom were treated in the intensive care unit. The results indicated that 60% of the isolates were tested for CZA susceptibility, with 97% showing susceptibility [23]. In a separate study conducted by Zhang et al., a total of 872 carbapenem-resistant *K. pneumoniae* isolates were collected from 36 different hospitals. The susceptibility rate to CZA was found to be 96.3% [24]. A 2023 study published in the USA analyzed 310 CRE isolates, determining that the CZA susceptibility rate for these isolates was 84.2% [25]. Additionally, a study conducted in Turkiye assessed the susceptibility of 42 carbapenem-resistant *K. pneumoniae* isolates to CZA, revealing that 91.4% of these isolates were susceptible [26]. In our study, all isolates were resistant to carbapenems, and the CZA susceptibility rate among these isolates was found to be 82.0%.

Silent antimicrobial resistance genes are bacterial genes present on plasmids or chromosomal DNA that do not exhibit phenotypic resistance to antibiotics. Silent (non-expressed) antibiotic resistance genes in bacteria may occur through different mechanisms. Various mutations in the resistance gene region, alterations/deletions within the promoter region, and insertion of insertion sequence elements can lead to inactivation/activation of drug resistance genes [27]. In a study mentioning the silent NDM gene, whole genome sequencing was performed on 5 *K. pneumoniae* isolates that carried the NDM gene but were susceptible to carbapenems, and it was found that all of these isolates had deletions in the upstream region of the NDM gene. It has been stated that this deletion inhibits the expression of the resistance gene and leads to phenotypic susceptibility to carbapenems. In vitro bactericidal tests and mouse infection model results performed in the same study consistently demonstrated that silent NDM in *K. pneumoniae*, despite susceptibility to carbapenems, was associated with failure of antibiotic treatment and recurrence of bacterial infection [28]. In a study that included carbapenem-resistant *K. pneumoniae* isolates, one of the five isolates carrying both the OXA-48 and NDM genes was found to be susceptible to CZA [26]. In a different study, the NDM gene was detected in 3 *K. pneumoniae* strains, and one of them was found to be susceptible to CZA [29]. In both of these studies, we could not find any explanation regarding CZA susceptibility in the presence of NDM. In our study, 3 isolates were susceptible to CZA even though they had the NDM gene. The NDM genes in these isolates may not be expressed, but since we could not perform whole genome sequencing, we cannot make a clear judgment on this issue.

Similarly, our study found varying susceptibility results based on the presence of different resistance genes for both CZA and MEV (Table 2). In studies involving *K. pneumoniae* isolates that carry the NDM gene, CZA resistance was typically found; however, few studies reported CZA susceptibility [30]. In our study, 3 of the 19 isolates carrying the NDM gene were susceptible to CZA. These discordant isolates were confirmed by repeat PCR and antibiotic susceptibility studies.

In a study conducted in the USA that examined various β-lactam/β-lactamase inhibitor combinations in 310 CRE isolates, the susceptibility to MEV was found to be 81.9% [25]. Another study in the USA reported that 15 out of 16 (94%) CRE isolates were susceptible to MEV [31]. Additionally, a study in Italy investigated the efficacy of MEV on carbapenem-resistant *K. pneumoniae* isolates. In this study, 18 KPC-producing isolates were subjected to susceptibility testing, and 66.6% were found to be susceptible to MEV [32]. We could not locate any other studies on MEV susceptibility in Gram-negative bacteria from our country. Notably, the MEV susceptibility rate in our study is relatively low at 10.1%, especially when compared to findings from other countries. Additionally, an analysis of the distribution of carbapenemase genes in various studies conducted in our country, including ours, reveals that the dominant gene is OXA-48, while the positivity rate for KPC is comparatively low [10]. Based on these findings, it is not surprising that our study showed low susceptibility to MEV. In our study, 8 isolates that were susceptible to MEV contained only the KPC gene, while 1 isolate had the OXA-48 gene alone. The SENTRY study published in 2021 included participants from 31 countries. Within this study, the OXA-48-like gene was identified in 354 *Enterobacterales* isolates, with *K. pneumoniae* accounting for 89.8% (318) of these cases. Notably, the metallo-β-lactamase gene was not detected in 315 of the 354 *Enterobacterales* isolates, and 99.0% of these 315 isolates were found to be susceptible to CZA, with 49.8% also showing susceptibility to MEV. Although MEV resistance is typically expected when the OXA-48 gene is present, isolates in which gene resistance was not reflected in the phenotype were also detected in other studies [33,34]. However, in these studies, we could not find an interpretation for the detected unexpected situation. The resistance gene may not be expressed in the isolate in our study that carries the OXA-48 gene and is susceptible to MEV.

In a multicenter study conducted across 36 countries, primarily in Europe, carbapenem resistance genes were identified in 1203 carbapenem-resistant *K. pneumoniae* isolates. The rates of these genes varied by country, with the following results: 31.5% were KPC, 25.8% OXA-48, 7.7% NDM, 5.7% VIM, and 29.3% represented other types [35]. In a study of 87 carbapenem-resistant *K. pneumoniae* strains analyzed in Bosnia and Herzegovina, carbapenem resistance genes were identified in 85 isolates, which represents 97.7% of the samples, using PCR testing. The OXA-48 gene was found in 83 isolates (95.4%), while both the KPC and NDM genes were detected in all isolates [36]. A study conducted in Turkiye examined a total of 112 isolates of carbapenem-resistant *Escherichia coli* and *K. pneumoniae* from 18 different centers. The research aimed to investigate the presence of carbapenemase genes in these isolates. The results revealed that 68.7% of the isolates carried the OXA-48 carbapenemase gene, 16.1% carried the NDM gene, 10.7% carried the KPC gene, and 4.5% carried the VIM resistance gene. In a separate study conducted in Turkiye, researchers examined a total of 100 *K. pneumoniae* isolates obtained from clinical samples of hospitalized patients between 2015 and 2017. The findings revealed that 81.05% of the isolates carried the OXA-48 gene, 38.9% carried the NDM gene, 9.47% carried the KPC gene, and 1.05% carried the VIM gene [10]. In our study, the most common gene was OXA-48 (79.8%), but NDM (21.4%) and KPC (17.9%) genes were also detected.

The co-production of different carbapenemases within the same isolate is commonly observed. Various studies have documented the co-production of combinations such as OXA-48 + NDM, OXA-48 + VIM, OXA-48 + KPC, KPC + NDM, and VIM + KPC [9,10,37,38,39]. In our study, the most frequently detected gene combinations were OXA-48 + NDM (9 cases), followed by OXA-48 + KPC (5 cases), OXA-48 + KPC + NDM (1 case), and KPC + NDM (1 case).

The in vitro antibiotic susceptibility results for 89 isolates reveal that the most effective treatment against carbapenem-resistant *K. pneumoniae* isolates in our hospital is CZA, with a susceptibility rate of 82.0%. This finding aligns with previous studies conducted in our country, which identified OXA-48 as the predominant carbapenemase gene present in our hospital. The high sensitivity rate is not surprising, given that CZA is specifically effective against OXA-48 [10,38]. Among the carbapenemase genes examined, OXA-48 alone was detected in more than half of the isolates (56 cases), all of which were susceptible to CZA. Additionally, at least one other carbapenemase from a different group—NDM, KPC—was found in 15 out of 89 cases of *K. pneumoniae*. Of these cases, 46.7% were susceptible to CZA.

MEV is known to be effective against KPC-producing bacteria but is not expected to work against OXA-48-producing bacteria [14]. In our study, only 20.2% (18 isolates) of the *K. pneumoniae* did not have the OXA-48 gene. When we examined the effectiveness of MEV against these 18 isolates, we found that it was effective in vitro against 8 of them. Among the 10 isolates resistant to MEV, 9 were found to carry the NDM gene, while only 1 isolate had the KPC gene. This discordant isolate was confirmed by repeat PCR and antibiotic susceptibility studies. Additionally, decreased expression or mutations in the porins present in the outer membrane of Gram-negative bacteria, as well as plasmid-encoded AmpC enzymes, may contribute to reduced susceptibility to carbapenems [40,41]. This specific isolate, identified only as KPC but resistant to MEV, may possess other resistance mechanisms contributing to its resistance.

All *K. pneumoniae* isolates included in our study were found to be resistant to three carbapenems: ertapenem, meropenem, and imipenem, in vitro. When we examined the effectiveness of β-lactam/β-lactamase inhibitor combinations in these isolates, we found that the most effective combination was CZA, with a susceptibility rate of 82.0%. This was followed by MEV; however, its susceptibility rate was significantly lower at 10.1%. The susceptibility rates for piperacillin/tazobactam and ceftolozane/tazobactam, which are included in the automated system we used for antibiotic susceptibility testing, were determined to be 0% and 2.2%, respectively. Based on these findings, CZA appears to be more effective than MEV, piperacillin/tazobactam, and ceftolozane/tazobactam in the treatment of carbapenem-resistant *K. pneumoniae* isolates at our hospital. Our study is the first to examine the in vitro sensitivity of MEV in *K. pneumoniae* isolates from Turkiye.

This study has several limitations. First, it was conducted in a single-center setting, which may limit the generalizability of the findings to other healthcare institutions with different epidemiological profiles and resistance patterns. Second, the sample size was relatively small, with only 89 carbapenem-resistant *Klebsiella pneumoniae* isolates included in the analysis. A larger and more diverse sample set from multiple centers would enhance the robustness of the findings. Third, since we did not perform clonal analysis, we could not determine whether the isolates were epidemiologically related or represented independent strains. Fourth, while molecular methods were used to detect carbapenemase genes, whole genome sequencing was not performed. This limits the ability to identify potential mutations, plasmid-mediated resistance mechanisms, or silent resistance genes that may influence phenotypic antibiotic susceptibility results. Additionally, the study did not include clinical outcome data, which would have provided insights into the real-world effectiveness of the tested β-lactam/β-lactamase inhibitor combinations. Future studies should address these limitations by incorporating multicenter data, larger sample sizes, whole genome sequencing, and clinical outcome analyses to provide a more comprehensive understanding of resistance mechanisms and therapeutic options.

## 5. Conclusions

The emergence of resistance to ceftazidime/avibactam and meropenem/vaborbactam significantly reduces the treatment options available for managing multidrug-resistant *K. pneumoniae*, leading to increased morbidity and mortality rates. These antibiotics are essential for treating infections caused by carbapenemase-producing bacteria; however, the development of resistance presents a serious challenge for infection management. Therefore, it is crucial to continuously monitor resistance mechanisms, evaluate their clinical implications, and implement comprehensive strategies to ensure the responsible use of these important antibiotics.

## Figures and Tables

**Table 1:** Distribution of resistance genes in 89 *K. pneumoniae* isolates.

Resistance Gene	No of Isolates
Total	89
OXA-48	56
OXA-48 + NDM	9
KPC	9
NDM	8
OXA-48 + KPC	5
OXA-48 + KPC + NDM	1
KPC + NDM	1

**Table 2:** Ceftazidime/avibactam and meropenem/vaborbactam susceptibility results according to resistance genes.

	Resistant		Susceptible	
Resistance Gene	CZA (n:15)	MEV(n:80)	CZA (n:73)	MEV(n:9)
OXA-48	-	55	56	1
OXA-48 + NDM	7	9	2	-
KPC	-	1	9	8
NDM	7	8	1	-
OXA-48 + KPC	-	5	5	-
OXA-48 + KPC + NDM	1	1	-	-
KPC + NDM	1	1	-	-

## Data Availability

All data obtained and analyzed for this clinical study are available from the corresponding author upon reasonable request.

## Supplementary Materials

The following supporting information can be downloaded at: https://www.mdpi.com/article/10.3390/pathogens14030220/s1, Table S1: Antibiotic susceptibility results and resistance gene distributions of 89 K. pneumoniae isolates.

## Abbreviations

The following abbreviations are used in this manuscript:

EUCAST	European committee on antimicrobial susceptibility testing
KPC	Klebsiella pneumoniae carbapenemase
MALDI-TOF	Matrix-assisted laser desorption ionization-time of flight
MEV	Meropenem/vaborbactam
NDM	New-Delhi metallo-β-lactamase
PCR	Polymerase chain reaction
VIM	Verona integron-encoded metallo-β-lactamase
