# Supplementary material for: Are New β-Lactam/β-Lactamase Inhibitor Combinations Promising Against Carbapenem-Resistant K. pneumoniae Isolates?
Source: Pathogens. 2025 Feb 24;14(3):220. doi: 10.3390/pathogens14030220 (PMC11944681; doi:10.3390/pathogens14030220)
Supplement: Supplementary file 1 [file pathogens-14-00220-s001.zip › pathogens-3478004-supplementary.pdf]

Supplementary Table S1. Antibiotic susceptibility results and resistance gene distributions of 89 K. pneumoniae isolates.

Isolate number	Resistance genes	Susceptibility results			
		ceftazidime avibactam	meropenem vaborbactam	ceftolozane tazobactam	piperacillin tazobactam
1	KPC	S	R	R	R
2	OXA48	S	S	R	R
3	OXA48 + NDM	R	R	R	R
4	NDM	R	R	R	R
5	OXA48	S	R	R	R
6	OXA48 + KPC + NDM	R	R	R	R
7	OXA48	S	R	R	R
8	NDM	R	R	R	R
9	OXA48 + NDM	R	R	R	R
10	OXA48	S	R	R	R
11	OXA48 + NDM	R	R	R	R
12	OXA48	S	R	R	R
13	OXA48	S	R	R	R
14	NDM	R	R	R	R
15	OXA48	S	R	R	R
16	OXA48	S	R	R	R
17	OXA48 + KPC	S	R	R	R
18	OXA48 + KPC	S	R	R	R
19	OXA48	S	R	R	R
20	OXA48 + NDM	R	R	R	R
21	OXA48	S	R	R	R

22	OXA48	S	R	R	R
23	KPC	S	S	R	R
24	OXA48	S	R	R	R
25	OXA48	S	R	R	R
26	KPC	S	S	R	R
27	OXA48	S	R	R	R
28	OXA48	S	R	R	R
29	OXA48 + NDM	S	R	R	R
30	OXA48	S	R	R	R
31	OXA48	S	R	R	R
32	OXA48	S	R	R	R
33	OXA48	S	R	R	R
34	OXA48	S	R	R	R
35	OXA48	S	R	S	R
36	OXA48 + NDM	S	R	R	R
37	OXA48	S	R	R	R
38	OXA48	S	R	R	R
39	NDM	R	R	R	R
40	OXA48	S	R	R	R
41	OXA48	S	R	R	R
42	KPC	S	S	R	R
43	OXA48	S	R	R	R
44	OXA48	S	R	R	R
45	OXA48	S	R	R	R
46	NDM	R	R	R	R
47	OXA48	S	R	R	R

48	OXA48 + KPC	S	R	S	R
49	OXA48	S	R	R	R
50	OXA48	S	R	R	R
51	OXA48 + NDM	R	R	R	R
52	OXA48	S	R	R	R
53	KPC	S	S	R	R
54	OXA48	S	R	R	R
55	OXA48	S	R	R	R
56	OXA48	S	R	R	R
57	OXA48	S	R	R	R
58	OXA48	S	R	R	R
59	NDM	S	R	R	R
60	OXA48	S	R	R	R
61	OXA48	S	R	R	R
62	KPC + NDM	R	R	R	R
63	OXA48	S	R	R	R
64	OXA48	S	R	R	R
65	KPC	S	S	R	R
66	OXA48	S	R	R	R
67	KPC	S	S	R	R
68	NDM	R	R	R	R
69	OXA48	S	R	R	R
70	OXA48	S	R	R	R
71	OXA48 + KPC	S	R	R	R
72	KPC	S	S	R	R
73	OXA48 + NDM	R	R	R	R

74	OXA48	S	R	R	R
75	OXA48	S	R	R	R
76	OXA48 + NDM	R	R	R	R
77	OXA48 + KPC	S	R	R	R
78	OXA48	S	R	R	R
79	OXA48	S	R	R	R
80	OXA48	S	R	R	R
81	NDM	R	R	R	R
82	OXA48	S	R	R	R
83	OXA48	S	R	R	R
84	OXA48	S	R	R	R
85	OXA48	S	R	R	R
86	KPC	S	S	R	R
87	OXA48	S	R	R	R
88	OXA48	S	R	R	R
89	OXA48	S	R	R	R
